# Impact of systemic inflammation on gastric cancer outcomes

**DOI:** 10.1371/journal.pone.0174085

**Published:** 2017-03-30

**Authors:** Xuechao Liu, Shangxiang Chen, Jianjun Liu, Dazhi Xu, Wei Li, Youqing Zhan, Yuanfang Li, Yingbo Chen, Zhiwei Zhou, Xiaowei Sun

**Affiliations:** 1 Sun Yat-sen University Cancer Center; State Key Laboratory of Oncology in South China; Collaborative Innovation Center for Cancer Medicine, Guangzhou, China; 2 Department of Gastric and Pancreatic Surgery, Sun Yat-sen University Cancer Center, Guangzhou, China; University of North Carolina at Chapel Hill School of Medicine, UNITED STATES

## Abstract

**Background:**

The prognostic value of neutrophil-lymphocyte ratio (NLR) and Glasgow Prognostic Score (GPS) has been extensively validated in various cancers. We aimed to examine the usefulness of a combination of NLR and GPS (named CNG) for predicting survival outcomes in patients after curative resection for gastric cancer (GC).

**Methods:**

We retrospectively analyzed the records of 1056 patients who underwent curative resection as initial treatment for GC from October 2000 to September 2012. The preoperative CNG was calculated as follows: patients with hypoalbuminemia (< 35 g/L), elevated C-reactive protein (> 10 mg/L), and elevated NLR (≥ 2) were allocated a score of 3; patients with two, one, or no abnormal values were allocated a score of 2, 1, or 0, respectively.

**Results:**

The NLR and GPS were the only inflammatory variables independently associated with overall survival (OS) in multivariate analysis. When they were replaced by CNG in multivariate analysis, CNG was independently associated with OS (hazard ratio [HR] for CNG 1 [1.367, 95% CI: 1.065–1.755; *P* = 0.014], CNG 2 [1.887, 95% CI: 1.182–3.011; *P* = 0.008], and CNG 3 [2.224, 95% CI: 1.238–3.997; *P* = 0.008]; *P* = 0.020). In stage-matched analysis, the prognostic significance was still maintained in stage I-III (*P* = 0.002, *P* = 0.042, and *P* < 0.001, respectively). In addition, 5-year survival rates ranged from 92% (stage I) to 35% (stage III) and from 65%(CNG 0) to 18%(CNG 3) with tumor-nodes-metastasis (TNM) stage or CNG alone. However, the combination of TNM and CNG stratified 5-year survival rates from 98% (TNM I, CNG 0) to 12% (TNM III, CNG 3).

**Conclusion:**

The preoperative CNG is a novel predictor of postoperative survival, and the combination of CNG and TNM effectively stratifies outcomes in patients after curative resection for GC.

## Introduction

Gastric cancer (GC) is the second most frequent cause of cancer-related death worldwide and affects approximately one million people annually[1–3]. Despite great improvements in diagnosis and treatment, the long-term survival of GC patients remains unsatisfactory and may be related to the relatively late stage of diagnosis [4–6]. Therefore, it is important to determine prognostic factors that can distinguish high-risk patients that require tailored treatment. Up to now, the widely accepted tumor–nodes–metastasis (TNM) system depends on a postoperative histological specimen. Hence, there have been continuing efforts to explore preoperative prognostic factors that will permit more accurate patient stratification and improve clinical decision-making.

It is increasingly recognized that the systemic inflammatory response plays an important role in the development and progression of cancer [7, 8]. It is also of interest that several inflammation-based prognostic scores, such as the Glasgow Prognostic Score (GPS), neutrophil-lymphocyte ratio (NLR), platelet-lymphocyte ratio (PLR), combination of platelet count and neutrophil-lymphocyte ratio (COP-NLR), and Prognostic Index (PI), have prognostic value for many types of cancer including GC[9–13]. Of these, the GPS, an inflammation-based prognostic score based on serum C-reactive protein and albumin levels, has been repeatedly reported to have prognostic value in GC [14, 15]. In addition, the NLR, a combination of circulating neutrophil and lymphocyte counts, has also been demonstrated as a promising independent prognostic factor in GC [16]. Furthermore, we hypothesized that an integrated indicator, named the CNG (combination of NLR and GPS), might comprehensively reflect the balance of host inflammatory status.

The aims of this retrospective study were to investigate the prognostic value of several inflammation-based prognostic scores, especially the CNG, and to validate whether the combination of CNG and TNM effectively stratifies outcomes for patients after curative resection for GC.

## Materials and methods

### Study population

A total of 1056 patients with GC who underwent D2 gastrectomy with R0 resection at the Cancer Center of Sun Yat-sen University between October 2000 and September 2012 were enrolled. The study was approved by the Research Ethics Committee at the Cancer Center of Sun Yat-sen University, and written informed consent was obtained.

All patients had histologically confirmed stage I-III gastric adenocarcinoma depending on postoperative histological specimen. Tumors were staged using the seventh edition of the American Joint Committee on Cancer (AJCC) TNM staging system[17]. After surgery, patients with stage II or III GC and no significant comorbidities precluding chemotherapy use received primarily 5-fluorouracil-based adjuvant chemotherapy by discussion at a multidisciplinary meeting. Patients that met all the following eligibility criteria were included in the analysis: (1) no neoadjuvant chemotherapy or radiotherapy, (2) entire set of clinicopathological and follow-up data regarding potential prognostic factors, (3) no recurrent gastric cancer, remnant gastric cancer, or other synchronous malignancy, (4) no acute infections or other inflammatory conditions in the two weeks prior to surgery.

The following data were evaluated: age, sex, preoperative routine laboratory measurements, postoperative tumor characteristics, and survival times. The preoperative blood sample was collected in the week before surgery. Papillary and moderately differentiated types of GC were categorized as the well-differentiated group, and signet ring cell, mucinous, and undifferentiated types were categorized as the poorly-differentiated group[18]. Complete blood counts, physical examinations, serum tumor marker measurements, dynamic CT examinations, and gastroscopy were performed every 3 months during the first 2 years after surgery and every 6 months thereafter. The end of follow-up was the date of last follow-up (June 2015) or death from all causes. Overall survival (OS) was defined as the interval between the date of surgery and the date of death from all causes or last follow-up.

### Calculation of biomarkers

The GPS was calculated as previously described. Patients with both an C-reactive protein level >10 mg/L and an albumin level <35 g/L were assigned a score of 2. Patients with only one or neither of these abnormalities were assigned a score of 1 or 0, respectively[19]. The NLR and PLR were defined as the absolute neutrophil count and platelet count divided by the absolute lymphocyte count, respectively[20]. Based on previous studies, the COP-NLR was calculated as follows: patients with an elevated platelet count (>300 × 10^9^/L) and an elevated neutrophil-lymphocyte ratio (>3) were assigned a score of 2. Patients with one or no abnormal value were assigned a score of 1 or 0, respectively[21]. The PI was calculated as follows: patients who had both a C-reactive protein level >10 mg/L and a white blood cell count >11× 10^9^/L were assigned a score of 2. Patients with only one or neither of these abnormalities were assigned a score of 1 or 0, respectively[22]. The CNG was defined as follows: patients with hypoalbuminemia (< 35 g/L), elevated C-reactive protein (> 10 mg/L) and NLR (≥ 2) were assigned a score of 3, and patients with two, one, or no abnormal value were assigned a score of 2, 1, or 0, respectively.

### Statistical analysis

As reported by other authors, the inflammation–based prognostic scores GPS, COP–NLR, and PI were defined using widely accepted thresholds[19, 21, 22]. For NLR, PLR, and other continuous variables, the optimal cutoff values were calculated using the Youden index (sensitivity + specificity-1) by receiver operating characteristic (ROC) curve analysis. The Pearson Chi-squared test was used to determine the significance of differences. Survival analysis was performed using the Kaplan-Meier method and compared by the log-rank test. Variables that proved to be significant (*P* <0.05) in the univariate analysis were tested subsequently with a multivariate Cox proportional hazard model with the enter method. To evaluate the discriminatory ability of prognostic scores, the ROC curves were constructed to compare the areas under the curve (AUC) values. Two-sided *P* <0.05 was considered to be statistically significant. All the statistical analyses were performed using SPSS 19.0 software (IBM Corporation, Armonk, NY, USA).

## Results

A total of 1056 patients were included in the study (714 men and 342 women). The mean age of the patients was 58 years (range 19–89 years). Overall, there were 194 (18.4%) patients with stage I, 266 (25.2%) patients with stage II, and 596 (56.4%) patients with stage III GC. The median follow-up period was 33 months (range 1–97 months).

**Fig 1:**
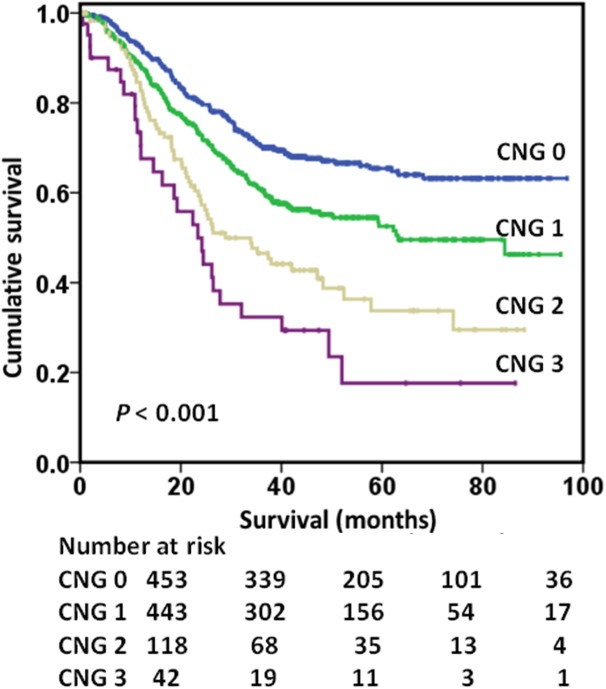
Kaplan–Meier survival curves stratified by the preoperative CNG (p <0.001). CNG = combination of neutrophil-lymphocyte ratio and Glasgow Prognostic Score.

There was a positive correlation between NLR and GPS (r = 0.221, *P* <0.001). Overall, 453 (42.9%) patients were classified as CNG 0, whereas 443 (42.0%), 118 (11.2%), and 42 (4.0%) patients were classified as CNG 1, CNG 2, and CNG 3, respectively. Patients classified as CNG 0 had a significantly longer mean survival (70.6 months) when compared with CNG 1 (59.7 months), CNG 2 (44.4 months) or CNG 3 (32.9 months) (*P* <0.001) patients. The OS rates of CNG 0, CNG 1, CNG 2 and CNG 3 patients were 63.2%, 46.3%, 29.5% and 17.6%, respectively (*P* <0.001; Fig 1). Therefore, CNG effectively classified patients into four independent groups.

Table 1 shows the results of the univariate and multivariate analysis. Multivariate analysis showed that age (*P* <0.001), histological grade (*P* = 0.006), tumor location (*P* = 0.001), TNM stage (*P* <0.001), NLR (*P* = 0.031), and GPS (*P* = 0.058) were independently associated with reduced OS, though GPS had a moderate prognostic significance. However, several other systemic inflammation–based prognostic scores, including PLR, PI, and COP-NLR, were not associated with survival. When NLR and GPS were replaced by CNG, multivariate analysis showed that CNG (hazard ratio [HR] for CNG 1 [1.367, 95% CI: 1.065–1.755; *P* = 0.014], CNG 2 [1.887, 95% CI: 1.182–3.011; *P* = 0.008], and CNG 3 [2.224, 95% CI: 1.238–3.997; *P* = 0.008]; *P* = 0.020; S1 Table) was an independent prognostic factor for OS along with age (*P* <0.001), histological grade (*P* = 0.006), tumor location (*P* = 0.001), TNM stage (*P* <0.001).

**Table 1:** Univariate and multivariate analyses in relation to overall survival.

	Univariate analysis		Multivariate analysis	
	HR (95% CI)	P-value	HR (95% CI)	P-value
Sex		0.669		
Female	1			
Male	0.955 (0.776, 1.177)			
Age (years)		<0.001		<0.001
< 60	1		1	
≥ 60	1.458 (1.197, 1.775)		1.506 (1.227, 1.848)	
Histological grade		0.007		0.006
Well differentiated	1		1	
Poorly differentiated	1.454 (1.107, 1.910)		1.490 (1.123, 1.978)	
Tumor size (cm)		<0.001		0.717
< 5	1		1	
≥ 5	1.893 (1.555, 2.305)		0.961 (0.773, 1.194)	
Tumor location		<0.001		0.001
Upper third	1		1	
Middle third	0.597 (0.456, 0.781)	<0.001	0.786 (0.594, 1.039)	0.091
Lower third	0.453 (0.362, 0.569)	<0.001	0.638 (0.502, 0.812)	<0.001
TNM stage		<0.001		<0.001
I	1		1	
II	2.634 (1.500, 4.626)	0.001	2.270 (1.285, 4.009)	0.005
III	10.588 (6.406, 17.501)	<0.001	8.810 (5.251, 14.781)	<0.001
PLR		0.008		0.241
< 130	1		1	
≥ 130	1.309 (1.074, 1.595)		0.866 (0.681, 1.101)	
PI		<0.001		0.381
0	1		1	
1	2.140 (1.074, 1.595)	<0.001	1.006 (0.669, 1.513)	0.977
2	2.464 (1.074, 1.595)	0.001	1.510 (0.770, 2.958)	0.230
COP-NLR		<0.001		0.523
0	1		1	
1	1.496 (1.215, 1.842)	<0.001	1.148 (0.890, 1.481)	0.287
2	1.614 (1.108, 2.353)	0.013	1.024 (0.654, 1.604)	0.916
NLR		<0.001		0.031
< 2	1		1	
≥ 2	1.544 (1.264, 1.886)		1.295 (1.024, 1.658)	
GPS		<0.001		0.058
0	1		1	
1	1.875 (1.463, 2.403)	<0.001	1.433 (0.976, 2.104)	0.067
2	2.940 (2.049, 4.218)	<0.001	1.889 (1.107, 3.224)	0.020

Abbreviations: HR = hazard ratio; CI = confidence interval; TNM = tumor-node-metastasis staging; PLR = platelet-lymphocyte ratio; PI = Prognostic Index; COP-NLR = combination of platelet count and neutrophil to lymphocyte ratio; NLR = neutrophil-lymphocyte ratio; GPS = Glasgow Prognostic Score.

In stage-matched analysis, the prognostic significance of CNG was maintained in stage I-III (*P* = 0.002, *P* = 0.042 and *P* <0.001, respectively; S1 Fig). However, the prognostic significance of NLR was only maintained in stage I (*P* = 0.002) and stage III (*P* = 0.010), but not in stage II (*P* = 0.233). The prognostic significance of GPS was only maintained in stage II (*P* = 0.039) and stage III (*P* <0.001), but not in stage I (*P* = 0.166).

To further evaluate the prognostic value of several systemic inflammation-based prognostic scores, ROC curves were performed to compare the AUC values. The CNG had a higher AUC value (0.60; P <0.001) than other scores, including the NLR, PLR, COP-NLR, PI, and GPS (0.56; 0.54; 0.56; 0.57; 0.57; S2 Fig).

The relationship between the CNG and clinicopathologic characteristics is shown in Table 2. An elevated CNG was associated with male patients (*P* = 0.032), larger tumor size (*P* <0.001), tumor location (upper third) (*P* <0.001), higher TNM stage (*P* <0.001), elevated PLR (*P* <0.001), elevated PI (*P* <0.001), and elevated COP-NLR (*P* <0.001).

**Table 2:** Relationship between the CNG and clinicopathologic characteristics.

	CNG 0	CNG 1	CNG 2	CNG 3	P value
	(n = 453)	(n = 443)	(n = 118)	(n = 42)	
Sex					0.032
Male	285	311	88	30	
Female	168	132	30	12	
Age (years)					0.052
< 60	258	225	53	19	
≥ 60	195	218	65	23	
Histological grade					0.472
Well differentiated	77	90	26	9	
Poorly differentiated	376	353	92	33	
Tumor size (cm)					< 0.001
< 5	324	249	32	13	
≥ 5	129	194	86	29	
Tumor location					< 0.001
Upper third	156	182	69	20	
Middle third	99	84	19	9	
Lower third	198	177	30	13	
TNM stage					< 0.001
I	106	76	10	2	
II	118	112	29	7	
III	229	255	79	33	
PLR					< 0.001
< 130	319	158	23	12	
≥ 130	134	285	95	30	
PI					< 0.001
0	449	398	25	0	
1	4	43	81	35	
2	0	2	12	7	
COP-NLR					< 0.001
0	385	235	27	9	
1	68	180	60	23	
2	0	28	31	10	

Abbreviations: CNG = combination of neutrophil-lymphocyte ratio and Glasgow Prognostic Score; TNM = tumor-node-metastasis staging; PLR = platelet-lymphocyte ratio; PI = Prognostic Index; COP-NLR = combination of platelet count and neutrophil-to-lymphocyte ratio.

Because CNG was significantly associated with several prognostic factors (tumor size, tumor location, and TNM stage), subgroup analyses were performed to more comprehensively examine the prognostic significance of CNG. It should be noted that, the prognostic significance was still maintained when stratified by tumor size (<5 cm: *P* <0.001; ≥5 cm: *P* = 0.001), tumor location (upper third: *P* >0.001; lower third: *P* <0.001), and TNM stage (I: *P* = 0.002; II: *P* = 0.042; III: *P* <0.001), though association with CNG was not significant in patients with middle third tumor location (*P* = 0.068).

**Fig 2:**
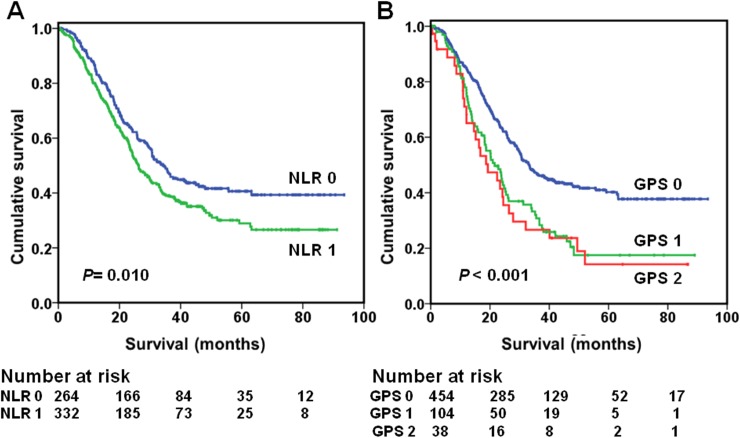
Overall survival based on the preoperative NLR (A) and GPS (B) in patients with stage III gastric cancer, respectively. NLR = neutrophil-lymphocyte ratio; GPS = Glasgow Prognostic Score.

**Fig 3:**
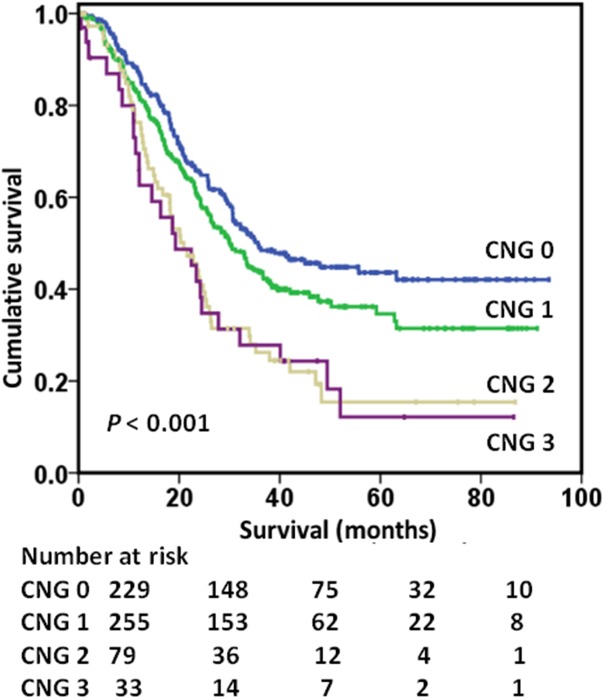
Relationship between CNG and TNM stage and OS of stage III gastric cancer patients (P <0.001). CNG = combination of neutrophil-lymphocyte ratio and Glasgow Prognostic Score; TNM = tumor–nodes–metastasis.

Table 3 shows the relationship between preoperative inflammation–based prognostic scores, TNM stage, and 5-year OS. Overall survival at 5 years ranged from 92% (stage I) to 35% (stage III), while 5-year survival rate varied from 62% (NLR < 2) to 47% (NLR ≥2), from 60% (GPS 0) to 24% (GPS 2) and from 65% (CNG 0) to 18% (CNG 3) with NLR, GPS, or CNG alone. When combined, 5-year survival rate varied from 98% (stage I, NLR < 2) to 29% (stage III, NLR ≥ 2) and from 93% (stage I, GPS 0) to 18% (stage III, GPS 1) (Fig 2). However, the combination of TNM stage and CNG stratified 5-year survival rate from 98% (stage I, CNG 0) to 12% (stage III, CNG 3) (*P* <0.001). As shown in Fig 3, the increased value of the combination of TNM stage and CNG on OS was evident for TNM stage III.

**Table 3:** Relationships between NLR, GPS, CNG, and 5-year OS.

	Stage I		Stage II		Stage III		Stage I-III	
	n	5-year OS	n	5-year OS	n	5-year OS	n	5-year OS
NLR	194	92 (3)	266	74 (4)	596	35 (2)	1056	55 (2)
< 2	109	98 (1)	132	77 (5)	264	41 (3)	505	62 (3)
≥ 2	85	83 (6)	134	70 (6)	332	29 (3)	551	47 (3)
GPS	194	92 (3)	266	74 (4)	596	35 (2)	1056	55 (2)
0	180	93 (3)	218	76 (4)	454	40 (3)	852	60 (2)
1	11	90 (10)	39	72 (10)	104	18 (5)	154	36 (5)
2	3	—	9	—	38	19 (7)	50	24 (7)
CNG	194	92 (3)	266	74 (4)	596	35 (2)	1056	55 (2)
0	106	98 (1)	118	78 (5)	229	44 (4)	453	65 (3)
1	76	83 (6)	112	75 (5)	255	35 (4)	443	53 (3)
2	10	—	29	63 (12)	79	15 (6)	118	34 (6)
3	3	—	7	—	33	12 (7)	42	18 (8)

The values are expressed as % (standard error); Survival is not calculated if n ≤ 10

Abbreviations: NLR = neutrophil-lymphocyte ratio; GPS = Glasgow Prognostic Score; CNG = combination of neutrophil-lymphocyte ratio and Glasgow Prognostic Score; OS = overall survival.

## Discussion

Although determinants of cancer progression and survival are multifactorial, the systemic inflammatory response is increasingly recognized as having a key role in carcinogenesis and disease progression[23]. Additionally, the mechanisms underlying cancer progression have remained a source of intense interest in recent years. In particular, the tumor microenvironment, orchestrated by inflammatory cells, has powerful effects on the carcinogenesis, proliferation, and migration[24]. In addition, by releasing proinflammatory cytokines, such as tumor necrosis factor-alpha (TNF-α), interleukin (IL)-6, and vascular endothelial growth factor (VEGF), tumor cells and tumor-associated leukocytes play a direct role in promoting proliferation and metastasis[25].

Over the past several decades, research has shown that some systemic inflammation-based scores represent an upregulation of the systemic inflammatory response[26–28]. Most notably, GPS has been regarded as a prognostic milestone in multiple cancer types, including GC[15]. Moreover, NLR has also been repeatedly reported to have prognostic value in various types of cancer[20]. A recent meta-analysis including 10 studies with a total of 2952 cases indicated that elevated NLR was a poor predictor for survival in GC[29].

In this study, we investigated the prognostic significance of several inflammation-based prognostic scores in a large cohort of patients undergoing curative resection of GC. Of these, only NLR and GPS were significantly associated with OS independent of TNM stage. To further refine prognostication and reflect the balance of host inflammatory status comprehensively, NLR and GPS were combined to generate a new inflammation-based prognostic score, named the CNG. In fact, A. Kinoshita et al suggested that the utility of the combination of a C-reactive protein-based prognostic score and white cell-based prognostic score for predicting survival of cancer patients should be validated in future trials[30].

A large incidental cohort analysis from Proctor MJ et al indicated that an inflammation-based prognostic score, combining high sensitivity C-reactive protein, albumin, and neutrophil count, had novel prognostic utility in cancer[31]. Similarly, we found that increased CNG was associated with larger tumor size and higher TNM stage. Therefore, it was parallel to tumor progression. Furthermore, an increased CNG was associated with male patients and upper third tumor, which may potentially reflect sex-specific and site-specific tumor heterogeneity. This should be validated in future multicenter randomized controlled studies. Of note, CNG had more potent prognostic value than other established prognostic scores in multivariate analyses, including PLR, PI, and COP-NLR. More importantly, we found its prognostic significance was maintained in stage I-III GC patients.

To further evaluate the prognostic ability, ROC curves were performed to compare the AUC values. It should be noted that the CNG had a higher AUC value than the NLR and GPS. In addition, we found that the combination of CNG and TNM stage increased the survival range compared to TNM or CNG alone. The combination of CNG and TNM stage also had a wider survival range than either NLR and TNM stage or GPS and TNM stage. Undoubtedly, CNG could identify more patients undergoing curative resection at higher risk of recurrence or metastasis than that afforded by TNM stage alone. Taken together, the data suggests that the CNG provides more potent prognostic value than the NLR and GPS. In line with our findings, a study of 12,119 cases from Proctor MJ et al confirmed that the addition of the neutrophil and platelet counts enhanced the prognostic value of the mGPS in cancer patients[32].

In clinical practice, preoperative CNG could help clinicians accurately identify patients with a high risk of tumor recurrence. Indeed, predicting which patients will have tumor recurrence after curative resection is difficult, especially patients with early-stage GC. Furthermore, patients with stage III GC usually develop early recurrence and metastasis. However, there has been no ideal prognostic indicator to provide information for further clinical treatment planning. It is of interest that CNG might serve as a powerful predictor of outcomes in stage I-III patients. Patients with an elevated CNG could benefit from closer monitoring and more aggressive surgical treatment (such as extended lymphadenectomy) and adjuvant chemotherapy, even in early-stage GC. The low cost, clinical availability, and reproducibility of a full blood count could make the CNG a valuable tool in the early decision-making process for patients with GC. Finally, it is increasingly appreciated that patients with an elevated systemic inflammatory response may benefit from targeted anti-inflammatory therapy and immunotherapy [33, 34]. Clinical research into the effects of nonsteroidal anti-inflammatory drugs for the prevention of various tumors is ongoing. Whether CNG can aid in selecting appropriate patients that may benefit from these therapies will be of considerable interest. Additional studies, especially prospective, multicenter, randomized controlled trials, are needed for validation.

The potential limitations of the present study are its confinement to a single center and its retrospective design. However, the surgical procedures (R0 resection plus D2 lymphadenectomy), laboratory tests, and follow-up were uniform during the entire study period. Although this study lacked disease-free survival and cancer-specific survival data, OS is the gold standard primary end point for evaluating cancer outcomes. Finally, different postoperative therapies may have had a confounding effect on our analysis of prognosis.

## Conclusion

The preoperative CNG, a novel inflammation-based prognostic score, is an independent prognostic factor of GC outcomes. Importantly, the combination of CNG and TNM effectively stratifies outcomes in patients after curative resection for GC. Measuring CNG and TNM in patients with GC may help improve clinical decision-making and ensure appropriate treatment.

## Supporting information

S1 Dataset (XLS)

S1 Fig Relationship between CNG and TNM stage and OS of stage I and stage II gastric cancer patients, respectively (P = 0.002; P = 0.042). CNG = combination of neutrophil lymphocyte ratio and Glasgow Prognostic Score; TNM = tumor–nodes–metastasis.
(TIF)

S2 Fig Comparison of the areas under the receiver operating characteristics curves for outcome prediction. CNG = combination of neutrophil-lymphocyte ratio and Glasgow Prognostic Score; NLR = neutrophil-lymphocyte ratio; PLR = platelet- lymphocyte ratio; PI = Prognostic Index; COP-NLR = combination of platelet count and neutrophil-to-lymphocyte ratio; GPS = Glasgow Prognostic Score.
(TIF)

S1 Table Univariate and multivariate analyses in relation to overall survival. Abbreviations: HR = hazard ratio; CI = confidence interval; TNM = tumor-node-metastasis staging; PLR = platelet-lymphocyte ratio; PI = Prognostic Index; COP-NLR = combination of platelet count and neutrophil-to-lymphocyte ratio; CNG = combination of neutrophil-lymphocyte ratio and Glasgow Prognostic Score.
(DOC)

## References

[1] Kamangar F, Dores GM, Anderson WF. Patterns of cancer incidence, mortality, and prevalence across five continents: defining priorities to reduce cancer disparities in different geographic regions of the world. Journal of clinical oncology: official journal of the American Society of Clinical Oncology. 2006;24(14):2137–50. Epub 2006/05/10. 16682732 10.1200/JCO.2005.05.2308

[2] Ferlay J, Soerjomataram I, Dikshit R, Eser S, Mathers C, Rebelo M, et al Cancer incidence and mortality worldwide: sources, methods and major patterns in GLOBOCAN 2012. International journal of cancer Journal international du cancer. 2015;136(5):E359–86. Epub 2014/09/16. 10.1002/ijc.29210 25220842

[3] Chen W, Zheng R, Zeng H, Zhang S. The incidence and mortality of major cancers in China, 2012. Chinese journal of cancer. 2016;35(1):73 Epub 2016/08/04. PubMed Central PMCID: PMCPmc4971631. 10.1186/s40880-016-0137-8 27484217 PMC4971631

[4] Sasako M, Sano T, Yamamoto S, Kurokawa Y, Nashimoto A, Kurita A, et al D2 lymphadenectomy alone or with para-aortic nodal dissection for gastric cancer. The New England journal of medicine. 2008;359(5):453–62. Epub 2008/08/02. 10.1056/NEJMoa0707035 18669424

[5] Rahman R, Asombang AW, Ibdah JA. Characteristics of gastric cancer in Asia. World journal of gastroenterology: WJG. 2014;20(16):4483–90. Epub 2014/05/02. PubMed Central PMCID: PMCPmc4000485. 10.3748/wjg.v20.i16.4483 24782601 PMC4000485

[6] Chen YS, Chen JG, Zhu J, Zhang YH, Ding LL. Long-term survival trends of gastric cancer patients between 1972 and 2011 in Qidong. Chinese journal of cancer. 2015;34(12):602–7. Epub 2015/10/21. PubMed Central PMCID: PMCPmc4615360. 10.1186/s40880-015-0058-y 26481511 PMC4615360

[7] Colotta F, Allavena P, Sica A, Garlanda C, Mantovani A. Cancer-related inflammation, the seventh hallmark of cancer: links to genetic instability. Carcinogenesis. 2009;30(7):1073–81. Epub 2009/05/27. 10.1093/carcin/bgp127 19468060

[8] Mantovani A, Allavena P, Sica A, Balkwill F. Cancer-related inflammation. Nature. 2008;454(7203):436–44. Epub 2008/07/25. 10.1038/nature07205 18650914

[9] Lorente D, Mateo J, Templeton AJ, Zafeiriou Z, Bianchini D, Ferraldeschi R, et al Baseline neutrophil-lymphocyte ratio (NLR) is associated with survival and response to treatment with second-line chemotherapy for advanced prostate cancer independent of baseline steroid use. Annals of oncology: official journal of the European Society for Medical Oncology / ESMO. 2015;26(4):750–5. Epub 2014/12/30. 10.1093/annonc/mdu587 25538172

[10] Koh CH, Bhoo-Pathy N, Ng KL, Jabir RS, Tan GH, See MH, et al Utility of pre-treatment neutrophil-lymphocyte ratio and platelet-lymphocyte ratio as prognostic factors in breast cancer. British journal of cancer. 2015;113(1):150–8. Epub 2015/05/30. 10.1038/bjc.2015.183 26022929 PMC4647546

[11] Proctor MJ, Morrison DS, Talwar D, Balmer SM, Fletcher CD, O'Reilly DS, et al A comparison of inflammation-based prognostic scores in patients with cancer. A Glasgow Inflammation Outcome Study. European journal of cancer (Oxford, England: 1990). 2011;47(17):2633–41. Epub 2011/07/05. 10.1016/j.ejca.2011.03.028 21724383

[12] Shiba H, Misawa T, Fujiwara Y, Futagawa Y, Furukawa K, Haruki K, et al Glasgow prognostic score predicts outcome after surgical resection of gallbladder cancer. World journal of surgery. 2015;39(3):753–8. Epub 2014/10/29. 10.1007/s00268-014-2844-0 25348884

[13] Zhang H, Zhang L, Zhu K, Shi B, Yin Y, Zhu J, et al Prognostic Significance of Combination of Preoperative Platelet Count and Neutrophil-Lymphocyte Ratio (COP-NLR) in Patients with Non-Small Cell Lung Cancer: Based on a Large Cohort Study. PloS one. 2015;10(5):e0126496 Epub 2015/05/08. PubMed Central PMCID: PMCPmc4423976. 10.1371/journal.pone.0126496 25950176 PMC4423976

[14] Hwang JE, Kim HN, Kim DE, Choi HJ, Jung SH, Shim HJ, et al Prognostic significance of a systemic inflammatory response in patients receiving first-line palliative chemotherapy for recurred or metastatic gastric cancer. BMC cancer. 2011;11:489 Epub 2011/11/23. PubMed Central PMCID: PMCPmc3226799. 10.1186/1471-2407-11-489 22103888 PMC3226799

[15] Wang DS, Ren C, Qiu MZ, Luo HY, Wang ZQ, Zhang DS, et al Comparison of the prognostic value of various preoperative inflammation-based factors in patients with stage III gastric cancer. Tumour biology: the journal of the International Society for Oncodevelopmental Biology and Medicine. 2012;33(3):749–56. Epub 2011/12/27. 22198641 10.1007/s13277-011-0285-z

[16] Hsu JT, Liao CK, Le PH, Chen TH, Lin CJ, Chen JS, et al Prognostic Value of the Preoperative Neutrophil to Lymphocyte Ratio in Resectable Gastric Cancer. Medicine. 2015;94(39):e1589 Epub 2015/10/02. 10.1097/MD.0000000000001589 26426635 PMC4616849

[17] Washington K. 7th edition of the AJCC cancer staging manual: stomach. Annals of surgical oncology. 2010;17(12):3077–9. Epub 2010/10/01. 10.1245/s10434-010-1362-z 20882416

[18] Ahn HS, Lee HJ, Hahn S, Kim WH, Lee KU, Sano T, et al Evaluation of the seventh American Joint Committee on Cancer/International Union Against Cancer Classification of gastric adenocarcinoma in comparison with the sixth classification. Cancer. 2010;116(24):5592–8. Epub 2010/08/26. 10.1002/cncr.25550 20737569

[19] Forrest LM, McMillan DC, McArdle CS, Angerson WJ, Dunlop DJ. Evaluation of cumulative prognostic scores based on the systemic inflammatory response in patients with inoperable non-small-cell lung cancer. British journal of cancer. 2003;89(6):1028–30. Epub 2003/09/11. PubMed Central PMCID: PMCPmc2376960. 10.1038/sj.bjc.6601242 12966420 PMC2376960

[20] Yodying H, Matsuda A, Miyashita M, Matsumoto S, Sakurazawa N, Yamada M, et al Prognostic Significance of Neutrophil-to-Lymphocyte Ratio and Platelet-to-Lymphocyte Ratio in Oncologic Outcomes of Esophageal Cancer: A Systematic Review and Meta-analysis. Annals of surgical oncology. 2015. Epub 2015/09/30. 10.1245/s10434-015-4869-5 26416715

[21] Ishizuka M, Oyama Y, Abe A, Kubota K. Combination of platelet count and neutrophil to lymphocyte ratio is a useful predictor of postoperative survival in patients undergoing surgery for gastric cancer. Journal of surgical oncology. 2014;110(8):935–41. Epub 2014/08/26. 10.1002/jso.23753 25146385

[22] Kasymjanova G, MacDonald N, Agulnik JS, Cohen V, Pepe C, Kreisman H, et al The predictive value of pre-treatment inflammatory markers in advanced non-small-cell lung cancer. Current oncology (Toronto, Ont). 2010;17(4):52–8. Epub 2010/08/11.PubMed Central PMCID: PMCPmc2913830. 10.3747/co.v17i4.567 PMC2913830 20697515

[23] Coussens LM, Werb Z. Inflammation and cancer. Nature. 2002;420(6917):860–7. Epub 2002/12/20. PubMed Central PMCID: PMCPmc2803035. 10.1038/nature01322 12490959 PMC2803035

[24] Wisastra R, Dekker FJ. Inflammation, Cancer and Oxidative Lipoxygenase Activity are Intimately Linked. Cancers. 2014;6(3):1500–21. Epub 2014/07/19. PubMed Central PMCID: PMCPmc4190552. 10.3390/cancers6031500 25037020 PMC4190552

[25] Balkwill F, Mantovani A. Inflammation and cancer: back to Virchow? Lancet. 2001;357(9255):539–45. Epub 2001/03/07. 10.1016/S0140-6736(00)04046-0 11229684

[26] Liu X, Sun X, Liu J, Kong P, Chen S, Zhan Y, et al Preoperative C-Reactive Protein/Albumin Ratio Predicts Prognosis of Patients after Curative Resection for Gastric Cancer. Translational oncology. 2015;8(4):339–45. Epub 2015/08/28. PubMed Central PMCID: PMCPmc4562973. 10.1016/j.tranon.2015.06.006 26310380 PMC4562973

[27] Hu B, Yang XR, Xu Y, Sun YF, Sun C, Guo W, et al Systemic immune-inflammation index predicts prognosis of patients after curative resection for hepatocellular carcinoma. Clinical cancer research: an official journal of the American Association for Cancer Research. 2014;20(23):6212–22. Epub 2014/10/02. 25271081 10.1158/1078-0432.CCR-14-0442

[28] Roxburgh CS, Salmond JM, Horgan PG, Oien KA, McMillan DC. Comparison of the prognostic value of inflammation-based pathologic and biochemical criteria in patients undergoing potentially curative resection for colorectal cancer. Annals of surgery. 2009;249(5):788–93. Epub 2009/04/24. 10.1097/SLA.0b013e3181a3e738 19387324

[29] Zhang X, Zhang W, Feng LJ. Prognostic significance of neutrophil lymphocyte ratio in patients with gastric cancer: a meta-analysis. PloS one. 2014;9(11):e111906 Epub 2014/11/18. PubMed Central PMCID: PMCPmc4234250. 10.1371/journal.pone.0111906 25401500 PMC4234250

[30] Kinoshita A, Onoda H, Imai N, Iwaku A, Oishi M, Tanaka K, et al The C-reactive protein/albumin ratio, a novel inflammation-based prognostic score, predicts outcomes in patients with hepatocellular carcinoma. Annals of surgical oncology. 2015;22(3):803–10. Epub 2014/09/06. 10.1245/s10434-014-4048-0 25190127

[31] Proctor MJ, McMillan DC, Horgan PG, Fletcher CD, Talwar D, Morrison DS. Systemic inflammation predicts all-cause mortality: a glasgow inflammation outcome study. PloS one. 2015;10(3):e0116206 Epub 2015/03/03. PubMed Central PMCID: PMCPmc4346265. 10.1371/journal.pone.0116206 25730322 PMC4346265

[32] Proctor MJ, Horgan PG, Talwar D, Fletcher CD, Morrison DS, McMillan DC. Optimization of the systemic inflammation-based Glasgow prognostic score: a Glasgow Inflammation Outcome Study. Cancer. 2013;119(12):2325–32. Epub 2013/04/12. 10.1002/cncr.28018 23575969

[33] Puntoni M, Marra D, Zanardi S, Decensi A. Inflammation and cancer prevention. Annals of oncology: official journal of the European Society for Medical Oncology / ESMO. 2008;19 Suppl 7:vii225–9. Epub 2008/09/20. 10.1093/annonc/mdn442 18790956

[34] Bertagnolli MM, Eagle CJ, Zauber AG, Redston M, Solomon SD, Kim K, et al Celecoxib for the prevention of sporadic colorectal adenomas. The New England journal of medicine. 2006;355(9):873–84. Epub 2006/09/01. 10.1056/NEJMoa061355 16943400

