# Supplementary figures and images for: Impact of systemic inflammation on gastric cancer outcomes
Source: PLoS One. 2017 Mar 30;12(3):e0174085. doi: 10.1371/journal.pone.0174085 (PMC5373584; doi:10.1371/journal.pone.0174085)

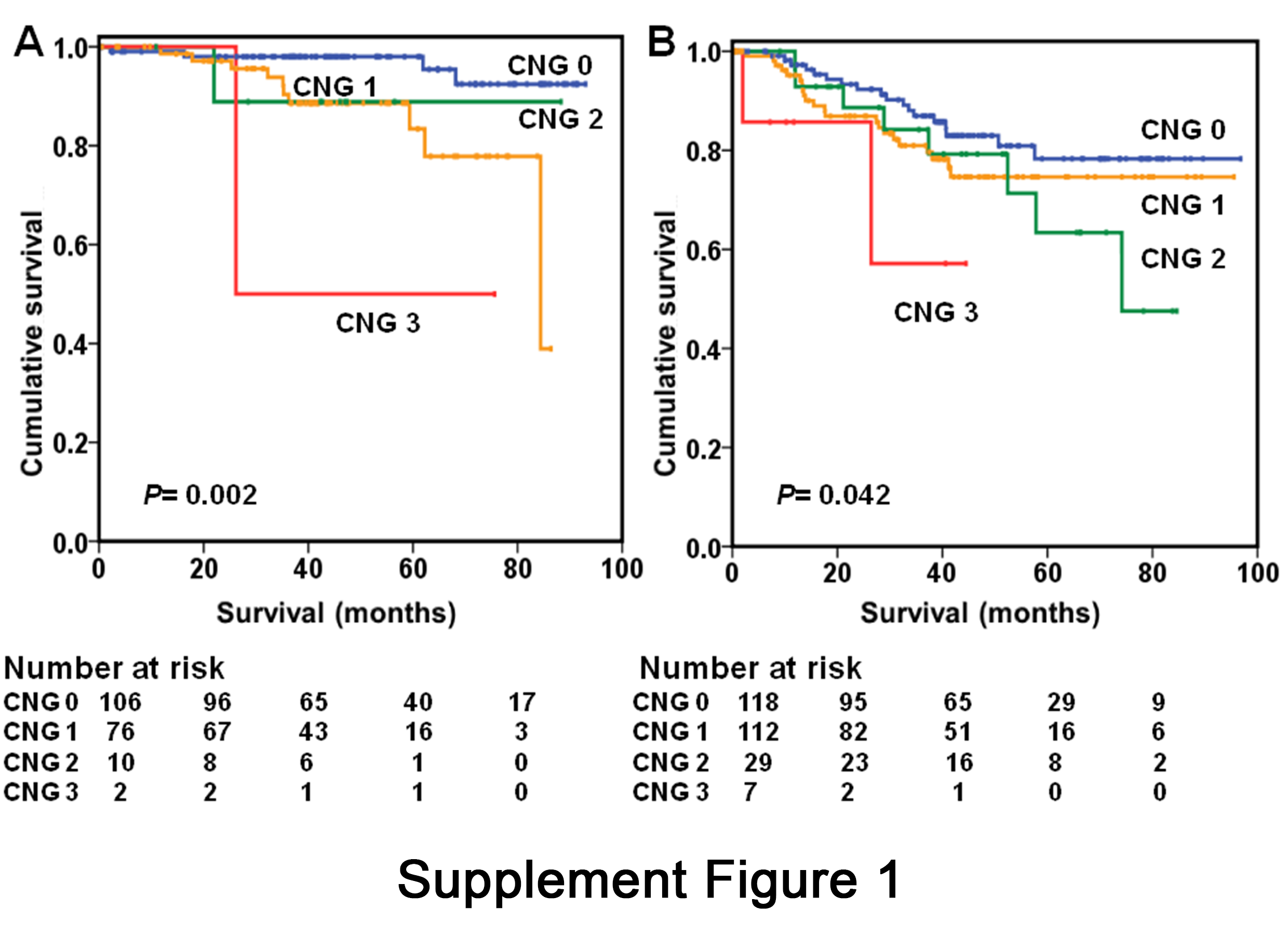

Supplement: S1 Fig — CNG = combination of neutrophil lymphocyte ratio and Glasgow Prognostic Score; TNM = tumor–nodes–metastasis. (TIF) [file pone.0174085.s002.tif]

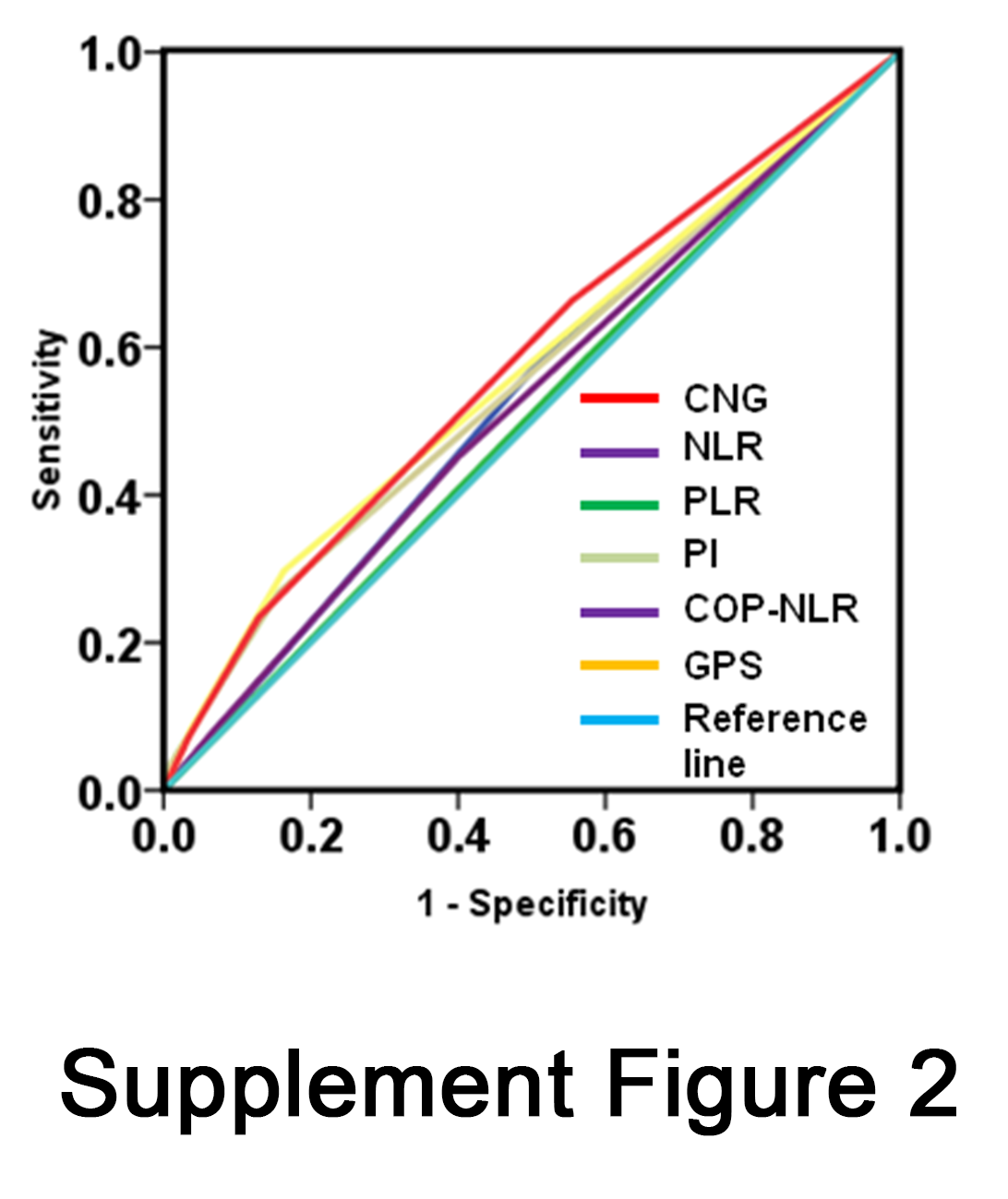

Supplement: S2 Fig — CNG = combination of neutrophil-lymphocyte ratio and Glasgow Prognostic Score; NLR = neutrophil-lymphocyte ratio; PLR = platelet- lymphocyte ratio; PI = Prognostic Index; COP-NLR = combination of platelet count and neutrophil-to-lymphocyte ratio; GPS = Glasgow Prognostic Score. (TIF) [file pone.0174085.s003.tif]
