# Supplementary material for: Impact of systemic inflammation on gastric cancer outcomes
Source: PLoS One. 2017 Mar 30;12(3):e0174085. doi: 10.1371/journal.pone.0174085 (PMC5373584; doi:10.1371/journal.pone.0174085)
Supplement: S1 Table — Abbreviations: HR = hazard ratio; CI = confidence interval; TNM = tumor-node-metastasis staging; PLR = platelet-lymphocyte ratio; PI = Prognostic Index; COP-NLR = combination of platelet count and neutrophil-to-lymphocyte ratio; CNG = combination of neutrophil-lymphocyte ratio and Glasgow Prognostic Score. (DOC) [file pone.0174085.s004.doc]

**S1 Table** Univariate and multivariate analyses in relation to overall survival

|  | Univariate analysis | Multivariate analysis |  |
| --- | --- | --- | --- |
|  | HR (95 % CI) P-value | HR (95 % CI) P-value |  |
| Sex | 0.669 |  |  |
| Female | 1.00 |  |  |
| Male | 0.955 (0.776, 1.177) |  |  |
| Age (years) | <0.001 | <0.001 |  |
| < 60 | 1.00 | 1.00 |  |
| ≥ 60 | 1.458 (1.197, 1.775) | 1.509 (1.231, 1.852) |  |
| Histological grade | 0.007 | 0.006 |  |
| Well differentiated | 1.00 | 1.00 |  |
| Poorly differentiated | 1.454 (1.107, 1.910) | 1.488 (1.121, 1.974) |  |
| Tumor size (cm) | <0.001 | 0.717 |  |
| < 5 | 1.00 | 1.00 |  |
| ≥ 5 | 1.893 (1.555, 2.305) | 0.961 (0.773, 1.194) |  |
| Tumor location | <0.001 | 0.001 |  |
| Upper third | 1.00 | 1.00 |  |
| Middle third | 0.597 (0.456, 0.781) <0.001 | 0.793 (0.600, 1.049) 0.105 |  |
| Lower third | 0.453 (0.362, 0.569) <0.001 | 0.643 (0.506, 0.818) <0.001 |  |
| TNM stage | <0.001 | <0.001 |  |
| I | 1.00 | 1.00 |  |
| II | 2.634 (1.500, 4.626) 0.001 | 2.276 (1.289, 4.018) 0.005 |  |
| III | 10.588 (6.406, 17.501) <0.001 | 8.861 (5.282, 14.867) <0.001 |  |
| PLR | 0.008 | 0.225 |  |
| < 130 | 1.00 | 1.00 |  |
| ≥ 130 | 1.309 (1.074, 1.595) | 0.862 (0.678, 1.096) |  |
| PI | <0.001 | 0.327 |  |
| 0 | 1.00 | 1.00 |  |
| 1 | 2.140 (1.074, 1.595) <0.001 | 1.057 (0.731, 1.528) 0.768 |  |
| 2 | 2.464 (1.074, 1.595) 0.001 | 1.602 (0.842, 3.047) 0.151 |  |
| COP-NLR | <0.001 | 0.597 |  |
| 0 | 1.00 | 1.00 |  |
| 1 | 1.496 (1.215, 1.842) <0.001 | 1.128 (0.879, 1.447) 0.344 |  |
| 2 | 1.614 (1.108, 2.353) 0.013 | 1.015 (0.650, 1.585) 0.950 |  |
| CNG | <0.001 | 0.020 |  |
| 0 | 1.00 | 1.00 |  |
| 1 | 1.513 (1.208, 1.895) <0.001 | 1.367 (1.065, 1.755) 0.014 |  |
| 2 | 2.369 (1.755, 3.197) <0.001 | 1.887 (1.182, 3.011) 0.008 |  |
| 3 | 3.557 (2.351, 5.383) <0.001 | 2.224 (1.238, 3.997) 0.008 |  |

Abbreviations: HR = hazard ratio; CI = confidence interval; TNM = tumour-node-metastasis staging; PLR = platelet lymphocyte ratio; PI = Prognostic Index; COP-NLR= combination of platelet count and neutrophil to lymphocyte ratio; CNG = combination of neutrophil lymphocyte ratio and Glasgow prognostic score.
